# An atypical presentation of sympathetic ophthalmia following chemical ocular burns

**DOI:** 10.1186/s12348-023-00348-z

**Published:** 2023-05-16

**Authors:** Pooja Bansal, Maninder Singh, Yashi Gupta, Nikhil Gotmare, Meenakshi Thakar, Ritu Arora

**Affiliations:** Guru Nanak Eye Centre, Maulana Azad Medical College, New Delhi, 110002 India

**Keywords:** Chemical ocular burns, Sympathetic ophthalmia, Steroids, Immunosuppressants

## Abstract

**Background:**

Sympathetic ophthalmia is a rare disease that can present as bilateral granulomatous uveitis after a penetrating trauma or surgery in one eye.

**Findings:**

We report a case of a 47-year-old male with history of decreased vision in the right eye, six months after sustaining severe chemical injury in the left eye. He was diagnosed with sympathetic ophthalmia and was treated with corticosteroids and long-term immunosuppressive therapy, leading to complete resolution of intraocular inflammation. Final visual acuity was 20/30 at one year of follow up.

**Conclusions:**

Sympathetic Ophthalmia following chemical ocular burns is extremely uncommon. It can present as a diagnostic and therapeutic challenge. It warrants early diagnosis and management.

## Introduction

Sympathetic ophthalmia (SO) is a rare disease that presents as bilateral granulomatous uveitis weeks to years, after a penetrating trauma or surgical intervention in one eye. The eye that sustains trauma or surgery is called the “exciting” eye and the fellow eye is said to be “sympathizing". Early diagnosis and timely management, can control the inflammation and prevent permanent vision loss. [1–4] Sympathetic ophthalmia induced by non-penetrating ocular trauma is extremely uncommon. [5–8] We here in present a case of SO following chemical ocular injury without clinical evidence of any ocular perforation, managed successfully with systemic steroids and immunosuppression.

## Case report

**Fig. 1:**
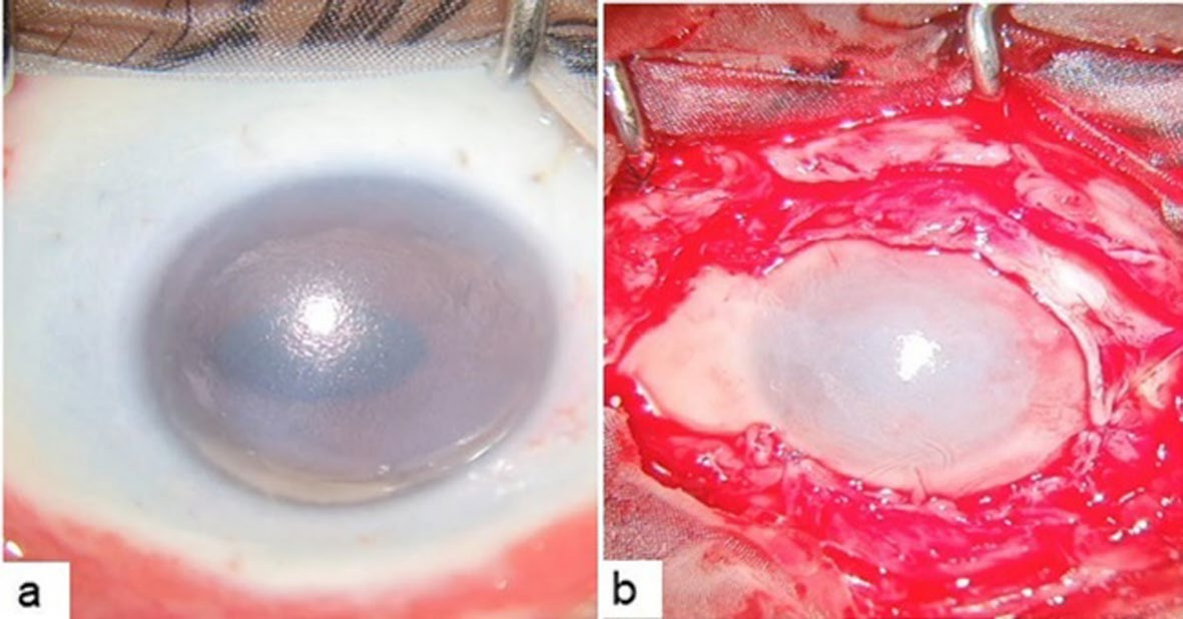
(**a**) Left eye showing severe chemical injury with 360 degree limbal ischemia, (**b**) after tenonplasty and amniotic membrane transplantation

A 47 year old Asian Indian male was referred to the retina clinic with sudden onset, painless diminution of vision and metamorphopsia in the right eye (OD) since one week. The patient got severe chemical burns in the left eye (OS) following an accidental fall of a toilet cleaner, six months ago, for which he underwent tenonplasty and amniotic membrane transplantation [Fig. 1a, b].

**Fig. 2:**
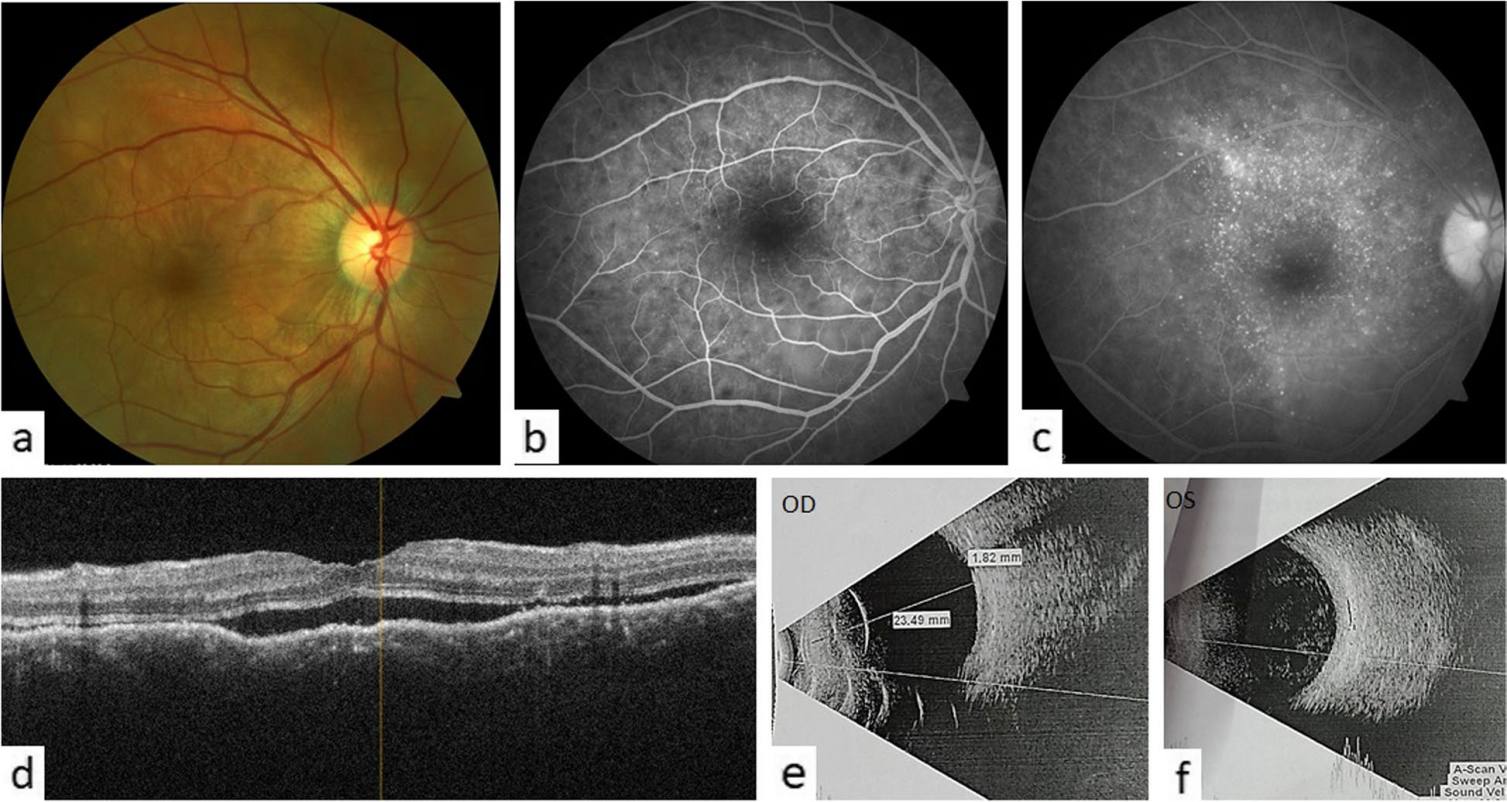
(**a**) Fundus picture of the right eye showed multiple round white subretinal lesions with pockets of subretinal fluid on the posterior pole, (**b**) FA showed multiple areas of pinpoint hypofluorescence in early phase, and (**c**) hyperfluorescence with pooling of the dye in late phase, (**d**) OCT macula showed diffuse bumpy choroidal thickening with sub retinal fluid, (**e**) and (**f**) USG B-scan revealed choroidal thickening in both the eyes with vitritis in the left (exciting) eye

**Fig. 3:**
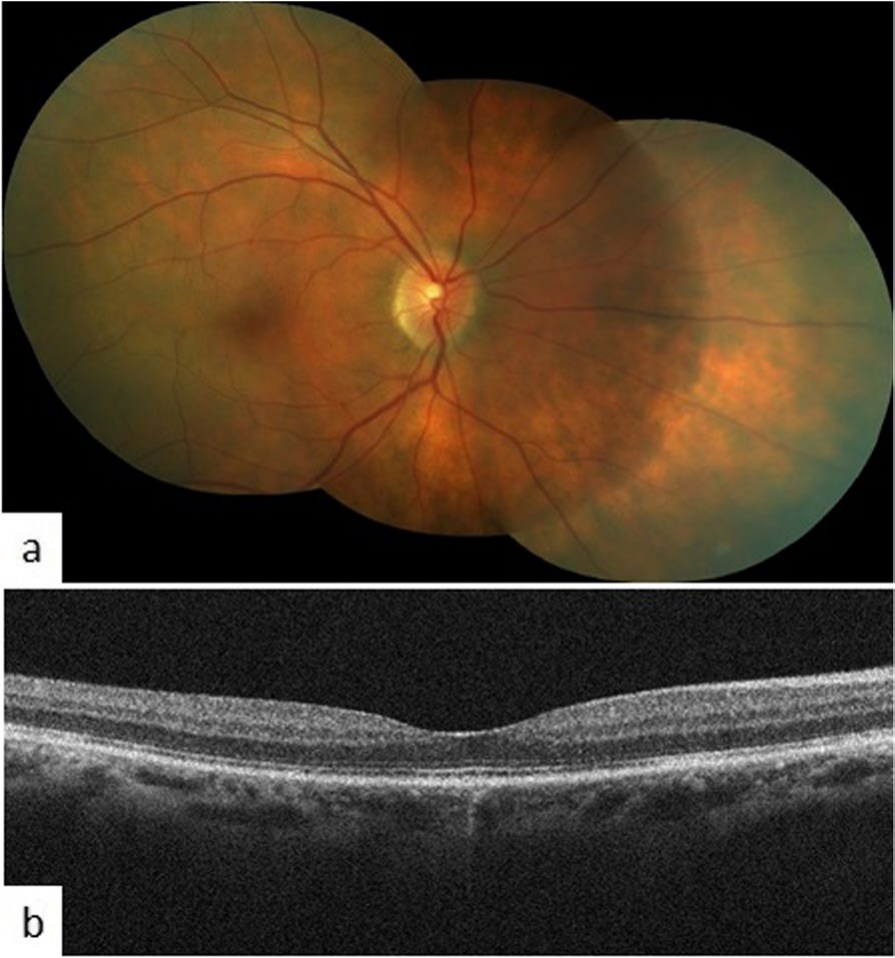
(**a**) BCVA was 6/9 with no signs of intraocular inflammation while the patient was on azathioprine 100 mg once a day at 9 months (**b**) OCT showed resolution of subretinal fluid and choroidal thickening

**Fig. 4:**
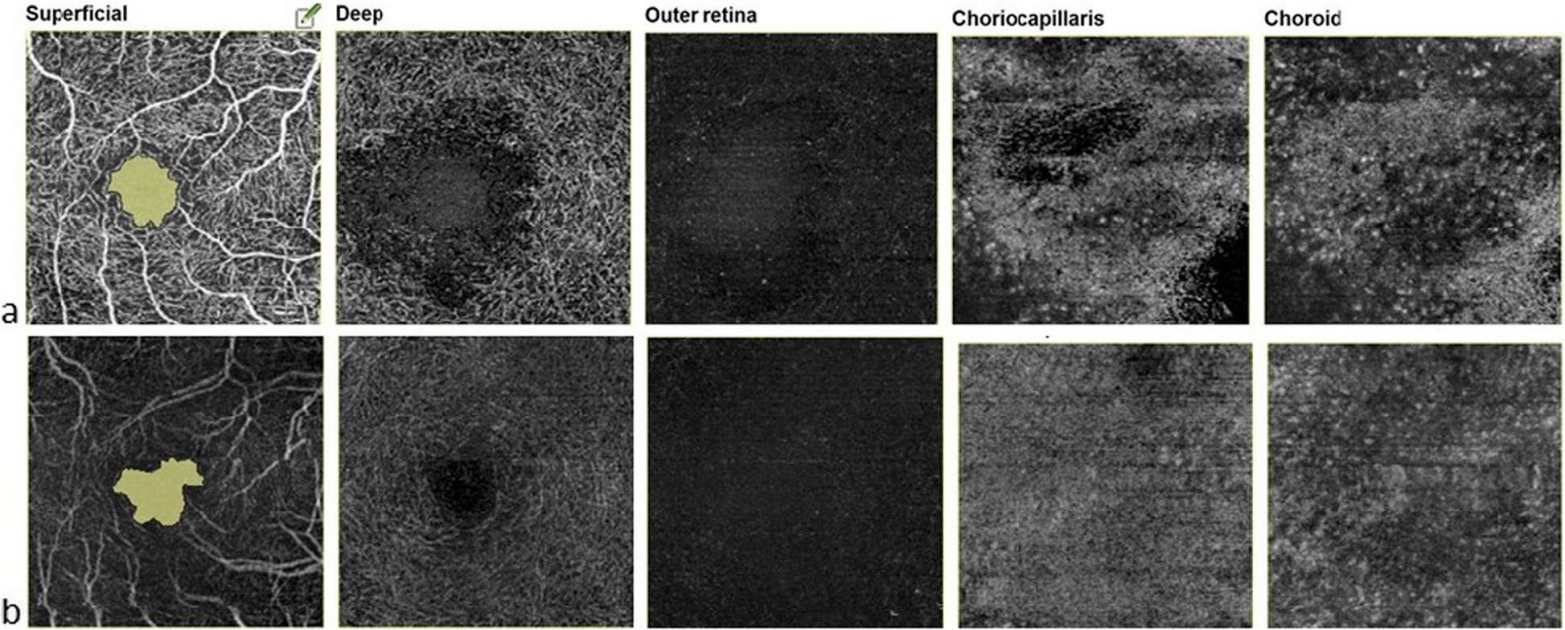
(**a**) OCTA at presentation showed distorted vasculature in superficial (SCP) and deep capillary plexus (DCP), the choroidal vasculature revealed pockets of flow voids (**b**) post treatment, SCP and DCP vascular pattern became organised; choroidal vasculature regained its granular pattern

At presentation, BCVA OD was 6/24 and OS had light perception. Intraocular pressures were 18 and 12 mm of Hg in OD and OS respectively. The anterior segment was quiet in the right eye. There was symblepharon formation with shortening of the palpebral fissure in the left eye. The cornea was opaque and vascularised. The pupil was horizontally distorted. Seidel test was negative. Anterior chamber and posterior segment details were not visible. Fundus examination OD revealed multifocal serous retinal detachments (SRDs) scattered across the posterior pole with a shallow detachment at the fovea (Fig. 2a). Fluorescein angiography (FA) showed multifocal pinpoint areas of hypo and hyperfluorescence over the posterior pole, with late dye pooling in the area of exudative detachment (Fig. 2b,c). The retinal pigment epithelium (RPE) undulations and sub retinal fluid (SRF) with diffuse thickening of the choroid was noticed on optical coherence tomography (OCT) macula (Fig. 2d). An ultrasound B scan of both eyes revealed thickened choroid with vitritis (Fig. 2e,f). Optical coherence tomography angiography (OCTA) macula revealed distorted vasculature in the superficial (SCP) and deep capillary plexus (DCP) of the retina, with multiple small areas of choriocapillaris flow voids, most likely corresponding to the areas of choriocapillaris ischemia (Fig. 4a). A diagnosis of sympathetic ophthalmia was made on the basis of history, clinical examination and ocular investigations. Systemic and neurological examinations were performed by an internist. Investigations such as Mantoux test, chest X-ray, urine and blood cultures, HIV and VDRL were done to rule out other systemic infections. Baseline laboratory tests were also obtained (FBS, CBC, LFT and KFTs). The patient was administered pulse therapy of intravenous methyl prednisolone (1 gm/day) for five days, followed by tapering doses of oral steroids ((initiated at 1 mg/kg/day; maximum dose of 70 mg/day). On tapering the oral steroids to 40 mg at 4 weeks, there was a relapse of posterior segment inflammation. The dose of oral steroids was again increased to 1 mg/kg/day; maximum dose 70 mg/day) and an immunosuppressive agent, azathioprine (1.5 mg/day) was added with slow tapering of the oral steroids subsequently. The patient gradually responded to the treatment and the exudative detachment resolved [Fig. 3a]. The OCT macula at 9 months, showed complete resolution of SRF and restoration of normal retinal and choroidal architectures (Fig. 3b). OCTA imaging also showed improvement of the retinal vasculature and flow voids of the choriocapillaris (Fig. 4b). On the last follow up, at 2 years, BCVA OD was 6/6 with a sunset glow fundus and no signs of intraocular inflammation while the patient was on azathioprine 25 mg once a day.

## Discussion

The diagnosis and management of sympathetic ophthalmia are often challenging, and usually based on the history and clinical examination findings. The clinical presentation and course of SO remain variable. It can manifest as panuveitis or posterior uveitis presenting with vitritis, choroiditis, disc edema, yellowish white nodules and multiple SRDs. The important risk factors for SO are penetrating trauma, delayed surgical repair after initial injury, and vitreoretinal surgery. [1–4] SO has also been reported following glaucoma filtering surgery, intraocular lens implantation, intravitreal injection, scleral buckling, non-penetrating procedures including laser or cryo-cyclodestructive procedures, and irradiation for choroidal melanoma. [5–9] There have been case reports of SO after acanthamoeba, fungal keratitis and therapeutic keratoplasty. [10–12] So far, there has been only one case of SO reported after chemical ocular burns where the exciting eye was found to have a corneal perforation with iris prolapse, which had initiated the event. [13] The development of SO in the absence of preceding penetrating trauma or surgery is very rare. [14].

Ocular antigens are normally sequestered within the blood retinal barrier (BRB), thus preventing exposure of these antigens and their detection by the systemic immune system. Though SO is considered an autoimmune disease where intraocular inflammation develops after sensitization to previously sequestered uveal antigens following a breach in the ocular barrier, the exact mechanism is still unclear. Zhang et al. reported two cases of SO caused by chemical burns in a case series of 22 eyes with SO following globe injury over a 5-year period. SO was attributed to the ocular perforation caused by the chemical injury, which led to incarceration of the uveal tract. [15] In our case, there was no visible ocular perforation, so we kept two hypotheses. The first hypothesis was that severe corneal burns with total limbal ischemia led to the release of pro-inflammatory mediators through a disturbed BRB to expose the retinal antigens, which subsequently led to inflammation in the sympathising eye. The second possibility was an occult globe perforation during chemical injury or AMT.

FA and SD-OCT provide valuable information for diagnosis and SD-OCT alone can often be used to monitor the disease activity. Newer imaging modalities like OCTA have also been found helpful in monitoring treatment response. [16–18].

Amniotic membrane transplantation with or without tarsorraphy is the recommended treatment for the injured eye with severe chemical ocular burns. The purpose of the treatment is to restore the corneal and conjunctival epithelial surfaces, as well as prevent ocular tissue melting. [19, 20]

The management of SO remains controversial. Some authors have recommended early enucleation of the injured eye to prevent SO, while others have not found it absolutely preventive. [21, 22] Lubin et al. reported that 74% of patients had 20/70 or better vision in the sympathizing eye if the exciting eye was enucleated within 2 weeks of onset, whereas if enucleation was performed after 6 months, only 50% of patients attained 20/70 or better vision. [23, 24] Treatment of SO involves initial control of the uveitis with high dose systemic corticosteroids in all patients, followed by long-term use of corticosteroid sparing immunosuppressive agents in the vast majority, especially for refractory and recurrent cases or for intolerable steroid-induced side effects. [25, 26] The visual prognosis of SO is relatively good if appropriate medical management is instituted in time.

## Conclusions

We report this rare presentation of sympathetic ophthalmia following chemical ocular burns with no clinical evidence of globe perforation in the exciting eye. Ophthalmologists should be mindful that sympathetic ophthalmia may result from non-penetrating ocular trauma. A high index of suspicion and regular monitoring of such patients may facilitate early recognition and treatment of SO, hence reducing visual morbidity.

## Data Availability

All data generated or analyzed during this study are included in this published article.
